# Hemoperitoneum among Pediatric Abdominal Trauma Patients Visiting in Emergency Department of a Tertiary Care Centre: A Descriptive Cross-sectional Study

**DOI:** 10.31729/jnma.7520

**Published:** 2023-01-31

**Authors:** Samjhana Basnet, Alok Pradhan, Sahil Bade, Grishma Sharma, Latika Giri, Yagya Ratna Shakya, Robin Man Karmacharya, Sushil Dahal, Sanu Krishna Shrestha

**Affiliations:** 1 Department of General Practice and Emergency Medicine, Dhulikhel Hospital, Kathmandu University Hospital, Kathmandu University School of Medical Sciences, Dhulikhel, Kavrepalanchowk, Nepal; 2 Kathmandu University School of Medical Sciences, Dhulikhel, Kavrepalanchowk, Nepal; 3 Department of Surgery, Dhulikhel Hospital, Kathmandu University Hospital, Kathmandu University School of Medical Sciences, Dhulikhel, Kavrepalanchowk, Nepal

**Keywords:** *blunt injuries*, *emergency medicine*, *focused assessment with sonography for trauma*

## Abstract

**Introduction:**

Pediatric abdominal trauma presents a major challenge for first-line responders in the Emergency Department for assessment and management. The Focused assessment sonography for trauma is a readily available, easy-to-use, and affordable tool for detecting hemoperitoneum during the initial assessment of trauma in the Emergency Department for adult traumatic patients. The aim of this study was to find the prevalence of hemoperitoneum among pediatric abdominal trauma patients visiting the Emergency Department of tertiary care centre through Focused assessment with sonography for trauma examination technique.

**Methods:**

This was a descriptive cross-sectional study conducted in the Emergency Department of a tertiary care hospital from 7 April 2019 to 7 April 2020. Among 413 pediatric trauma patients, 93 children (1 to 17 years) admitted to the Emergency Department who underwent focused assessment with sonography for trauma examination were included in the study. Ethical approval was obtained from the Institutional Review Committee (Approval number: 111/19). Convenience sampling was used. Point estimate and 90% Confidence Interval were calculated.

**Results:**

Among 93 children receiving focused assessment with sonography for trauma imaging in the Emergency Department with a history of blunt abdominal trauma, the prevalence of hemoperitoneum was 18 (19.34%) (12.61-26.09, 90% Confidence Interval).

**Conclusions:**

The prevalence of hemoperitoneum was similar to other studies conducted in a similar setting.

## INTRODUCTION

In middle and low-income countries, nine among 10 children are dying due to injuries among which abdominal trauma is still a leading cause of morbidity and mortality.^1^ Child typically has less fat to protect the intra-abdominal organs and it can be particularly difficult to elicit detailed history among young children or neurological deficit trauma children.^2^

Focused Assessment Sonography for Trauma (FAST) can be utilized to screen hemoperitoneum in trauma children. It is noninvasive, portable, cost-effective with zero radiation hazards and can be performed in the Emergency Department (ED).^3^ Contrast-enhanced computed tomography (CECT) is considered the gold standard for diagnosing hemoperitoneum, but it requires the moving of patients from the ED.^4^ FAST is widely established in adult trauma assessment and has eliminated DPL (Diagnostic Peritoneal Lavage) procedure.^5,6^ Through FAST, time management can be achieved and fatal outcomes caused by a missed diagnosis can be prevented.

This study aimed to find out the prevalence of hemoperitoneum among pediatric abdominal trauma patient through the focused assessment with sonography for trauma examination technique.

## METHODS

This was a descriptive cross-sectional study conducted in the ED of Dhulikhel Hospital, Kathmandu University Teaching Hospital from 7 April 2019 to 7 April 2020. The ethical approval was obtained from the Institutional Review Committee of Kathmandu University School of Medical Science, Dhulikhel Hospital (IRC-KUSMS Approval number: 111/19). A total of 413 pediatric trauma patients were identified from the medical record in the given time period. Out of which 93 pediatric patients were enrolled in the study i.e. children with age one to 17 years of age, diagnosis of abdominal injury and admission to ED with documentation of FAST examination, were selected. Whereas adults, with non-traumatic injury, penetrating injury, patients with abdominal CECT imaging findings before the arrival to the ED, unambiguous FAST scan, referral to another centre, discharged and declared dead were excluded from the study.

The sample size was calculated by using the following formula:


$$\begin{array}{ll} \text{n} & ={\text{Z}}^{2}\times \frac{\text{p}\times \text{q}}{{\text{e}}^{\text{2}}}\\ & =1.645^{2}\times \frac{0.50\times 0.50}{0.09^{2}}\\ & =84 \end{array}$$

Where,

n = minimum required sample size
Z = 1.645 at 90% Confidence Interval (CI)
p = prevalence is taken as 50% for maximum sample size calculation
q = 1-p
e = margin of error, 9%

The calculated sample size was 84. After adjusting the sample size for a 10% non-response rate, the final sample size was 93.

The data were abstracted using convenience sampling by the investigator and anonymity was maintained in all stages. The clinical data were collected from the record file of the ED. We included age, gender, mechanism of injury, emergency FAST report, CECT report and intraoperative findings, A "true positive'' was defined as when both the FAST (Showing free fluid in right upper quadrant view and showing free fluid in pelvic view) and CECT abdomen/ intraoperative findings indicated the presence of free fluid in the peritoneal cavity. A "true Negative'' was when both FAST and CECT abdomen findings were negative for free fluid. A "False Positive'' was when the FAST finding was positive but the CECT abdomen was negative for free fluid, and a "False negative'' was when the FAST finding was negative but the CECT abdomen finding was positive for free fluid.

Data were entered in an excel sheet and analyzed with IBM SPSS Statistics 20.0. Point estimate and 90% CI were calculated.

## RESULTS

Out of 93 pediatric patients who were admitted to ED for the management of abdominal trauma, 18 (19.35%) (12.61-26.09, 90% CI) patients showed hemoperitoneum on examination by focused assessment of sonography for trauma examination. There were 14 (77.77%) males and 4 (22.23%) females with a male-to-female ratio of 3.5:1. There were five children (27.77%) of age one to ten years of age and 13 (72.23%) children 10-17 years of age were having hemoperitoneum. The most common cause of traumatic injury in children was fall injury 12 (66.66%) (Table 1).

Among all traumatic hemoperitoneum presented to the centre, 16 constituted solid organ injury (88.89%) and two constituted hollow viscus injury (11.11%). Likewise, only 2 (11.11%) of the patients with blunt abdominal trauma presented within one hour of injury and the 16 (88.89%) presented after a golden hour of injury. The common site of free blood collection among those who had hemoperitoneum was the Spleenorenal fossa 13 (72.22%), followed by Morrison's space 11 (61.11%) and Pouch of douglas 10 (55.55%) (Table 1).

**Table 1:** Characteristics of patients (n = 18).

Characteristics		n (%)
Age	<10 years	5 (27.77)
	>10 years	13 (72.23)
Gender		
	Male	14 (77.77)
	Female	4 (22.23)
Mechanism of injury	Fall injury	12 (66.66)
	Other injuries	6 (33.34)
Intra-Abdominal injury	Solid	16 (88.89)
	Hollow viscus	2 (11.11)
Golden hour		
	Within 1 hour	2 (11.11)
	Above 1 hour	16 (88.89)
Free fluid		
	Splenorenal	13 (72.22)
	Morrison	11 (61.11)
	Pouch of Douglas	10 (55.55)

The most common intra-abdominal organ injured was the spleen 13 (16.66%) followed by liver 3 (16.66%). There were jejunum and kidney injury cases and each constitutes two in number (11.11%) (Table 2). Further, there was one ileum injury case which was found in intra-operative findings.

**Table 2:** Intra-abdominal organ involvement (n= 18).

Organ involvement	n (%)
Liver	3 (16.66)
Spleen	13 (72.22)
Jejunum	2 (11.11)
Ileum	-
Pancreas	1 (5.55)
Kidney	2 (11.11)

For all participants, the FAST examination technique resulted in a sensitivity of 94.7%, a specificity of 100%, a negative predictive value of 98.66%, and a positive predictive value of 100%.

## DISCUSSION

We found that the prevalence of hemoperitoneum in blunt abdominal trauma in the pediatric population is 19.35%. This finding is similar to the prospective study conducted among 108 children, in which hemoperitoneum was detected with the CECT abdomen examination technique and documented a prevalence of 24%.^7^ CECT is considered the gold standard for diagnosing free fluid (blood) in the abdominal cavity, but it requires moving patients from the ED which poses a significant risk in hemodynamically unstable patients and it is an expensive and invasive procedure.^8^

FAST is widely established in the ED management of adult trauma populations and has eliminated the need for performing DPL (Diagnostic Peritoneal Lavage) altogether.^5^ There are several studies showing that FAST in the pediatric population is as good as in adults for initial screening for hemoperitoneum in blunt abdominal trauma.^9^ Similarly, studies were conducted. in a pediatric population reported the accuracy of FAST to be 97%.^9,4^ These studies show that incorporating the FAST scan during the initial evaluation results in better clinical outcomes in children.^10^ In our study, it is found that 19.34% (18 patients) had true positive haemoperitoneum, one had false negative hemoperitoneum and 24% were true negative hemoperitoneum.

The present study indicates male preponderance in pediatric trauma. The majority of the pediatric patients enrolled in this study were males. Many studies have shown a higher injury rate in males than in females.^11-13^ This gender disparity in the pediatric population could be due to disproportionate risk-taking behaviour, perceptions of masculinity, exposure to potential risk factors like playing on the road, trees, construction sites etc.^14^ A study conducted in Eastern Nepal among 3958 pediatric trauma patients also found a disproportionately high percentage of males with traumatic injuries (78.4%).^15^

In this study, the most common mechanism of injury was also a fall injury followed by road traffic accidents. This finding correlates with the reports of several other studies.^15-17^ Several factors like evolving motor and cognitive skills in childhood, lack of adult supervision, lack of access to safe play spaces and opportunities, lack of preventive measures such as stair gates and guard rails, overcrowding, and awareness of fall risks among caregivers and rough play could contribute to the increased incidence of fall among children.^18^

A majority of abdominal injuries detected by the FAST examination technique were solid organ injuries. The result of the study advocates that a FAST positive result is higher in solid organ injury than hollow viscus injury. The most common organ injured was the spleen followed by the liver which is consistent with the studies.^19,20^

Studies have shown that point-of-care ultrasound (POCUS) applications like FAST allow physicians to rapidly obtain and interpret images and then apply those findings appropriately to the patient and the clinical context, which increases its relevance in resource-limited settings.^21^ Providing clinical care in the absence of diagnostic technologies like FAST also bears the risk of inappropriate treatment and missed diagnoses that can significantly influence health outcomes. These barriers result in both underdiagnosis and delayed diagnosis, resulting in increased morbidity and mortality.^21,22^ However, a randomized controlled trial among hemodynamically stable children who sustained blunt abdomen trauma did not significantly improve clinical care or decrease resource utilization as in adults which has shown that FAST examination during the initial evaluation resulted in decreased abdominal CT use, hospital lengths of stay, complications, and hospital charges.^23^

In cases of blunt abdominal trauma, even with solid organ injuries, conservative management has been practised along with operative management.^24^ A non-invasive tool like FAST is a better approach for the detection of injuries. Also, ultrasonography can be used in adjunct with other clinical and imaging parameters for the continuous evaluation of patients during conservative management. The proper use of FAST scans for the initial evaluation of such patients will help in the segregation of cases and might reduce patient costs in investigations.

There was one case with ileal injury, which did not have hemoperitoneum in the initial ultrasound examination but had gas under the diaphragm on the erect X-ray abdomen view. When a patient presents early in ED due to a hollow viscus injury there might be a lack of fluid collection and the injuries might be missed. The role of FAST in the evaluation of pneumoperitoneum has not yet been established. Sonographic signs like enhancement of peritoneal stripe and reverberation with a ring-down artefact starting from the peritoneum have been validated in previous studies.^25^ Further, Intraabdominal injuries in the pediatric population might be missed when USG is performed by a less proficient ED physician. Similarly, pediatric patients considered at high risk of injuries benefit from CECT even if the FAST was negative and vice versa.^10,26^ However, extensive training of ED physicians could improve sensitivity. Similarly, serial FAST examination technique in patients with negative initial FAST or adding other diagnostic adjuncts such as x-ray Abdomen erect and supine view or CECT abdomen may facilitate in detecting intra-abdominal injuries.^27,28^ The relatively small sample size of pediatric trauma patients and single-centre study design limits the generalization of findings to other contexts. Additionally, only admitted cases were included in the study and patients who were referred to other centres or who died were excluded. The retrospective nature of the study is a significant limitation as the initial FAST scan results were taken into account whereas repeat FAST scans whenever indicated were performed and could have had different results thus altering the results of the study.

## CONCLUSIONS

The prevalence of hemoperitoneum was similar to the other study in similar setting. Although they determined hemoperitoneum by using the CECT abdomen examination technique. Therefore, in low resources countries like Nepal, where CECT is not available in all settings or is expensive, portable ultrasonography techniques such as Focused Assessment with Sonography for Trauma examination technique can be utilized in the primary assessment of pediatric trauma victims to screen hemoperitoneum.
